# Intracellular Calcium Dysregulation: The Hidden Culprit in the Diabetes–Gout Nexus

**DOI:** 10.3390/biomedicines13112694

**Published:** 2025-11-02

**Authors:** Hongbin Shi, Yisi Shan, Kewei Qian, Ruofei Zhao, Hong Li

**Affiliations:** 1 Department of Endocrinology, Zhangjiagang TCM Hospital Affiliated to Nanjing University of Chinese Medicine, Zhangjiagang 215600, China; shb198705@outlook.com (H.S.); qiankewei@163.com (K.Q.); 2 Department of Neurology, Zhangjiagang TCM Hospital Affiliated to Nanjing University of Chinese Medicine, Zhangjiagang 215600, China; xssys333@163.com

**Keywords:** calcium homeostasis, type 2 diabetes, gout, insulin resistance, inflammasome

## Abstract

Type 2 diabetes and gout are both common metabolic disorders that frequently occur together. Research indicates that disturbances in intracellular calcium balance may be a key molecular factor linking the development of these two diseases. Calcium signaling disturbances promote the synergistic progression of both diseases through multiple pathways: In pancreatic β-cells, endoplasmic reticulum (ER) calcium imbalance triggers ER stress, mitochondrial dysfunction, and apoptosis, autophagy, and pyroptosis, leading to impaired insulin secretion. Concurrently, calcium overload exacerbates insulin resistance by disrupting insulin signal transduction in peripheral tissues, while hyperinsulinemia further inhibits uric acid excretion through activation of the renal URAT1 transporter, creating a vicious cycle. Additionally, calcium homeostasis dysregulation activates the NLRP3 inflammasome and promotes the release of pro-inflammatory cytokines, aggravating chronic low-grade inflammation, which further deteriorates β-cell function and peripheral metabolic disorders, collectively driving the pathological link between type 2 diabetes and gout. Although calcium channel modulators show potential in improving β-cell function and reducing inflammation, their clinical application faces challenges such as tissue-specific effects and a lack of high-quality clinical trials. We propose that intracellular calcium dysregulation serves as a central pathological amplifier in the diabetes–gout nexus. Future research on targeted calcium signaling interventions, guided by this integrative concept, may help overcome the therapeutic challenges in managing type 2 diabetes complicated by gout.

## 1. Introduction

Diabetes mellitus ranks as one of the most common metabolic conditions, presenting a major challenge to global health due to its links to mortality and disability [1,2,3]. According to the latest International Diabetes Federation (IDF) Atlas (11th edition, 2025), the global prevalence of diabetes continues to rise alarmingly, affecting approximately 537 million adults in 2025, with projections indicating this figure may reach 783 million by 2045 [4]. Type 2 diabetes accounts for the vast majority (>90%) of these cases, primarily driven by insulin resistance and dysfunction of β-cells. As type 2 diabetes progresses, it leads to various acute and chronic complications and is closely associated with another metabolic condition, gout. A meta-analysis shows that 16.70% of individuals with gout also have diabetes, with this figure rising to 20.70% in North America [5]. Additionally, a retrospective cohort study in the UK found that those with type 2 diabetes face a 48% greater risk of developing gout compared to non-diabetic individuals of the same age and gender [6]. Thus, type 2 diabetes and gout, both highly prevalent metabolic disorders, exhibit a significant comorbid relationship. This escalating comorbidity underscores the urgent need to elucidate the shared molecular pathways, among which intracellular calcium dysregulation has emerged as a critical but underexplored nexus. This review aims to synthesize the current understanding of calcium homeostasis in type 2 diabetes and gout, bridging fundamental mechanisms with clinical implications to identify novel therapeutic targets.

In this review, we synthesize current evidence to advance the hypothesis that intracellular calcium dysregulation acts as a keystone pathological mechanism and a central amplifier in the comorbidity of type 2 diabetes and gout. We will illustrate how calcium imbalance creates a self-reinforcing vicious cycle by simultaneously driving the core pathologies of both diseases—β-cell failure and insulin resistance in type 2 diabetes, and NLRP3-mediated inflammation in gout—thereby providing a unified perspective on this complex metabolic entanglement.

## 2. The Pathogenesis of Type 2 Diabetes Coexisting with Gout

Type 2 diabetes can disrupt the metabolism of uric acid through various pathways, contributing to the development of gout. One key factor is the impaired lipid metabolism seen in type 2 diabetes, which leads to higher concentrations of free fatty acids (FFAs). This increase promotes the synthesis of purines and the production of uric acid [7]. Additionally, the early stages of hyperinsulinemia linked to type 2 diabetes stimulate the Na^+^-H^+^ exchanger in the kidney’s proximal convoluted tubules. This activation enhances the reabsorption of uric acid by upregulating the urate transporter 1 (URAT1), resulting in elevated uric acid levels and the risk of gout [8,9]. Moreover, type 2 diabetes can trigger the polyol pathway, which boosts fructose production [10]. The significant ATP consumption during the metabolism of fructose increases the availability of purines, further worsening the likelihood of developing gout [11].

Conversely, gout can also play a role in the development and worsening of type 2 diabetes. Initially, the production of uric acid through xanthine oxidoreductase (XOR) is known to stimulate the renin–angiotensin–aldosterone system (RAS), which leads to increased levels of angiotensin II (Ang II) [12]. This rise in Ang II subsequently causes the generation of reactive oxygen species (ROS), resulting in oxidative stress and persistent low-grade inflammation. These detrimental processes directly hinder the functionality of pancreatic β-cells and worsen insulin resistance (IR), thus contributing to the onset of type 2 diabetes [13]. Secondly, during hyperuricemia episodes associated with gout, the activation of peroxisome proliferator-activated receptor-γ (PPAR-γ) in fat cells is inhibited. This suppression decreases the secretion of insulin-sensitizing hormones like adiponectin and increases the expression of monocyte chemoattractant protein-1 (MCP-1), both of which further intensify insulin resistance [14]. Furthermore, uric acid interferes with the insulin signaling pathway by attracting ectonucleotide pyrophosphatase/phosphodiesterase 1 (ENPP1) to the insulin receptor, thereby worsening the progression of type 2 diabetes [15]. In summary, there is a reciprocal relationship between type 2 diabetes and gout, where each condition aggravates the other, leading to a worsening of metabolic disorders.

**Figure 1:**
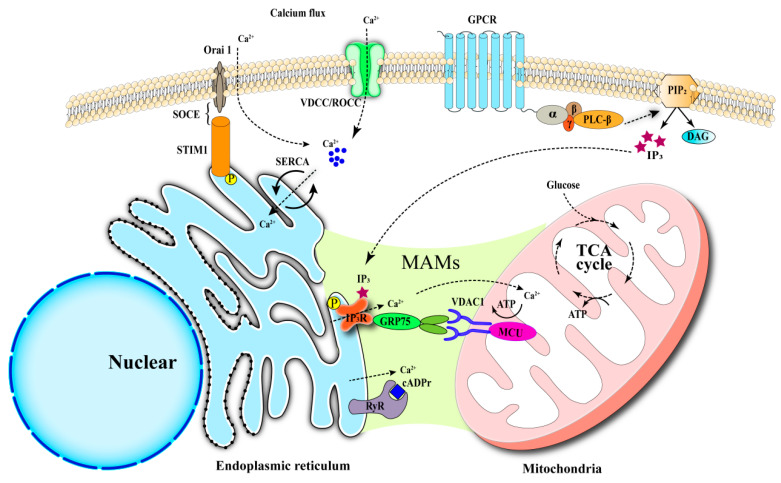
Core Mechanisms of Intracellular Calcium Homeostasis. (1) Calcium enters cells via plasma membrane channels (VDCCs, ROCCs, SOCE). (2) The endoplasmic reticulum (ER) acts as a major calcium store, regulated by SERCA (uptake) and IP3R/RyR (release). (3) Mitochondria uptake calcium via the MCU and MAMs to link calcium signaling with metabolism.

The regulation of calcium levels within cells is essential for proper cellular operations. The concentration of Ca^2+^ inside cells is carefully controlled by calcium channels in the plasma membrane, calcium reserves in the endoplasmic reticulum, and calcium cycling in mitochondria showed in Figure 1 and Table 1 [16]. The interaction between STIM1 and Orai1 at the plasma membrane enables store-operated calcium entry (SOCE), allowing calcium ions to flow into the cells. Calcium ions are stored in the endoplasmic reticulum through the action of the sarco/endoplasmic reticulum Ca^2+^-ATPase (SERCA) and are released via ryanodine receptors (RyR) and inositol 1,4,5-trisphosphate receptors (IP3Rs) [17,18,19,20]. Furthermore, calcium signaling is mediated through mitochondria-associated membranes (MAMs), a functional interface that coordinates signaling between the ER and mitochondria, as well as lysosomal contact sites to uphold calcium homeostasis and cellular functionality [21,22,23,24]. In pancreatic β cells, following the transport of glucose into the cells via glucose transporter 2 (GLUT2), an imbalance in the ATP/ADP ratio results in the closure of ATP-sensitive potassium (K^+^-ATP) channels, subsequently activating voltage-gated calcium channels (VGCC) to facilitate Ca^2+^ influx. This influx triggers calcium transients, thereby promoting insulin secretion [25,26,27,28]. Calcium ions function as second messengers, which play a crucial role in several key physiological processes, including lipid synthesis [29], energy metabolism [30], and programmed cell death [31]. Disruption of calcium homeostasis can lead to pancreatic β cell damage [32], activation of inflammatory responses [33], and impairment of insulin receptor signaling [34,35], thereby contributing significantly to the pathogenesis of various metabolic disorders such as obesity [36], diabetes [37], hyperuricemia [38], and liver diseases [39]. Consequently, the dysregulation of calcium homeostasis may represent a key molecular mechanism underlying the comorbidity of type 2 diabetes and gout.

## 3. Intracellular Calcium Homeostasis and Type 2 Diabetes

Disruption of intracellular calcium homeostasis is a critical factor contributing to β-cell damage and insulin resistance. Under pathological conditions, the imbalance in calcium homeostasis establishes a deleterious cycle with endoplasmic reticulum stress and the accumulation of inflammatory mediators. This cycle directly impairs β-cell function and inhibits insulin secretion while simultaneously exacerbating insulin resistance in peripheral tissues by disrupting insulin signal transduction. This bidirectional regulatory mechanism positions calcium homeostasis as a significant amplifier in the pathogenesis and progression of diabetes [52,53]. Clinical studies have demonstrated that insulin resistance and β-cell dysfunction increase the risk of developing diabetes by 3.2 times and 4.8 times, respectively. When both conditions coexist, the risk escalates to 35.9 times [54]. This non-linear increase in risk indicates a substantial synergistic pathogenic interaction between insulin resistance and β-cell dysfunction. From the perspective of disease progression, early-stage type 2 diabetes is marked by a reduction in insulin sensitivity within peripheral tissues. During this phase, β-cells sustain blood glucose homeostasis through compensatory proliferation. As insulin resistance progressively deteriorates, β-cells experience a prolonged state of overload, leading to successive pathological changes, including oxidative stress, endoplasmic reticulum stress, and chronic inflammation [55,56]. Throughout this process, an imbalance in calcium homeostasis emerges as a critical molecular event, driving multiple terminal damage pathways such as β-cell apoptosis, autophagy dysfunction, and ferroptosis, collectively advancing the progression of diabetes.

### 3.1. Intracellular Calcium Homeostasis and β-Cell Injury

The programmed death of pancreatic β-cells is orchestrated by a multitude of pathological factors acting in a coordinated manner. Notably, oxidative stress, endoplasmic reticulum stress, and mitochondrial stress exhibit significant molecular interactions with imbalances in calcium homeostasis [57,58,59]. As shown in Table 2, the programmed death of β-cells is characterized by multiple modalities, primarily encompassing apoptosis, regulated necrosis, and autophagy, among others. These modes of cell death form a complex regulatory network: lysosomal calcium release can induce ferroptosis by obstructing autophagic flux; activation of the AMPK-mTOR pathway inhibits autophagy while promoting β-cell apoptosis; and in a high-glucose environment, the inhibition of autophagy in INS-1 cells results in pyroptosis [60,61]. This multi-pathway, cross-interaction mechanism of cell death not only underscores the complexity of β-cell demise but also offers a novel molecular perspective for understanding the progressive functional decline of β-cells in diabetes.

#### 3.1.1. Apoptosis in Pancreatic β-Cells

##### Endoplasmic Reticulum-Related Apoptosis

**Figure 2:**
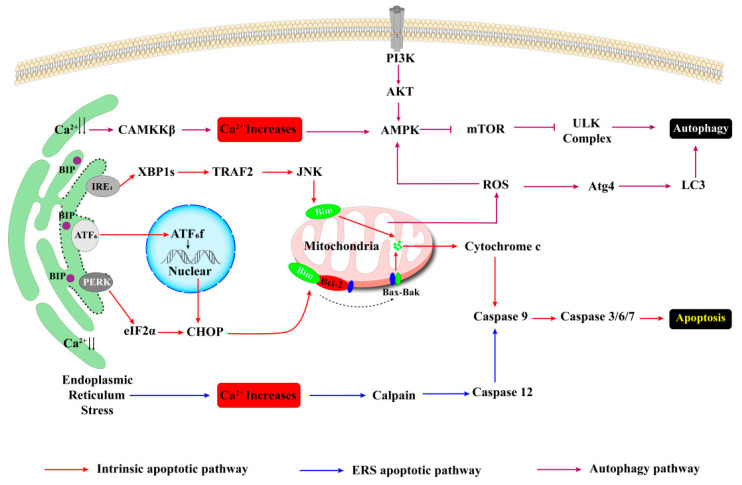
Regulation of Apoptosis and Autophagy by Calcium Homeostasis and Key Signaling Pathways. ER stress-induced calcium overload activates both intrinsic and ER-specific apoptotic pathways. Mitochondrial ROS production from calcium overload promotes autophagy via CAMKKβ-AMPK signaling. Autophagy and apoptosis are interconnected, forming a feedback loop that determines cell fate.

Multiple calcium ion channels play a crucial role in maintaining intracellular calcium homeostasis and the balance of endoplasmic reticulum stress. As highly specialized endocrine cells, β-cells exhibit heightened sensitivity to pathological stimuli such as inflammation, metabolic disturbances, and oxidative stress, which can initiate ER stress responses. In response to these stressors, the BiP/GRP78 chaperone system attempts to restore cellular homeostasis by activating the three branches of the unfolded protein response (UPR): IRE1α, PERK, and ATF6. Nonetheless, chronic ER stress can ultimately lead to β-cell apoptosis showed in Figure 2 [66,67,68,69,70,71,72]. A critical factor in this process is the disruption of intracellular calcium homeostasis due to abnormal SERCA function, which serves as a primary trigger for β-cell apoptosis. Research indicates that in mouse models of obesity induced by a high-fat diet, sustained high glucose levels impair SERCA function in β-cells, leading to ER calcium depletion. This depletion subsequently activates the UPR pathways: PERK/eIF2α/CHOP, ATF6-XBP1, and IRE1α/JNK/XBP1, culminating in β-cell apoptosis [40,41]. Subsequent investigations have demonstrated that intracellular calcium overload, resulting from SERCA dysfunction, primarily facilitates the formation of the DR5/FADD/caspase-8 apoptotic complex through the activation of the NF-κB/TLR-4 signaling pathway, thereby exacerbating β-cell apoptosis [42]. Various calcium release channels exhibit a concerted action in modulating β-cell apoptosis. The calcium homeostasis of the endoplasmic reticulum is sustained by calcium pumps (SERCA) and calcium release channels (IP3Rs/RyRs). Experimental evidence indicates that while the inhibition of RyR or IP3R in isolation has a limited effect on β-cell viability, simultaneous inhibition of both channels can significantly diminish cell death and alleviate endoplasmic reticulum stress [43,44,62]. Notably, the calcium release effects mediated by IP3Rs and RyRs synergize with the SERCA inhibitor thapsigargin, collectively resulting in endoplasmic reticulum calcium depletion and promoting β-cell apoptosis [43]. These findings highlight the crucial function of IP3Rs and RyRs in mediating β-cell apoptosis associated with SERCA dysfunction. Abnormalities in the store-operated calcium entry (SOCE) pathway represent a significant contributor to the exacerbation of calcium homeostasis disorders. Activation of the SOCE pathway occurs upon depletion of calcium reserves in the endoplasmic reticulum. Research has demonstrated that in the presence of SERCA inhibition, the CaMKII/Pyk2 pathway facilitates the tyrosine phosphorylation of STIM1, which subsequently forms a stable complex with Orai1, resulting in sustained calcium influx. The compromised function of SERCA prevents the alleviation of intracellular calcium overload, ultimately leading to β-cell apoptosis via the IRE1-JNK pathway [45]. This mechanism elucidates the detrimental cyclical relationship between disturbances in calcium signaling and endoplasmic reticulum stress.

Beyond the classical caspase-dependent apoptosis pathway, calcium signaling can influence the fate of β-cells by modulating the dynamic equilibrium of apoptotic proteins. A study by Liu revealed that Chlorogenic Acid (CA) significantly downregulates the expression of pro-apoptotic proteins such as Bax and caspase-3/9 by decreasing the cytoplasmic calcium concentration. Simultaneously, it upregulates the level of the anti-apoptotic protein Bcl-2. As a result, this leads to an improvement in glucose-stimulated insulin secretion (GSIS) and a notable inhibition of β-cell apoptosis [73].

##### Mitochondria-Associated Apoptosis

Mitochondrial dysfunction mediated by calcium homeostasis imbalance is one of the crucial mechanisms underlying β-cell apoptosis [74,75]. Empirical evidence suggests that aberrant calcium signaling can prompt the opening of the mitochondrial permeability transition pore (PTP), resulting in the dissipation of the mitochondrial membrane potential, thereby triggering downstream apoptotic cascades [76,77]. Under conditions of hyperglycemia or hyperlipidemia, calcium depletion in the endoplasmic reticulum occurs. Calcium signals are aberrantly transferred from the endoplasmic reticulum to mitochondria via the IP3Rs-GRP75-VDAC-MCU structural coupling. This process not only initiates the mitochondrial unfolded protein response (UPRmt) but also elicits mitochondrial stress [46,78,79]. This state of mitochondrial stress is manifested as the collapse of the mitochondrial membrane potential, impairments in ATP synthesis, and the explosive generation of reactive oxygen species (ROS). Excessive ROS further disrupts the balance of mitochondrial dynamics, inducing abnormal fission or fusion, thereby establishing a vicious cycle encompassing electron transport chain defects, bioenergetic dysregulation, and calcium homeostasis imbalance [80]. It is worth noting that upon treatment with thapsigargin, SERCA in β-cells is inhibited. This results in a decrease in calcium uptake and an increase in calcium release by the endoplasmic reticulum. Initially, this causes a transient elevation in the mitochondrial membrane potential [43], presumably representing a compensatory response of mitochondria to the sudden surge in cytoplasmic calcium ion concentration. However, over an extended period, marked mitochondrial depolarization becomes evident. When IP3R is concurrently activated at this stage, it accelerates the process of mitochondrial membrane potential collapse. Eventually, this leads to the irreversible opening of the PTP, the release of pro-apoptotic factors such as cytochrome c, and the activation of apoptotic effector proteases like caspase-9, thereby instigating the programmed death of β-cells.

#### 3.1.2. Autophagy in Pancreatic β-Cells

In the pathogenesis of type 2 diabetes, the functional impairment of pancreatic islet β-cells is notably associated with the dynamic regulation of autophagic activity. Moreover, autophagy and apoptosis exhibit a bidirectional regulatory interplay. Moderately activated autophagy can play an anti-apoptotic role by selectively removing damaged organelles, thereby maintaining cellular homeostasis. In contrast, over-activated autophagy may induce autophagic cell death, synergistically facilitating the apoptotic process [81,82].

Intracellular calcium signals intricately regulate the autophagy process via multiple pathways (Figure 2). Primarily, the release of reactive oxygen species (ROS) induced by mitochondrial stress can activate the lysosomal TRPML1 channel, leading to the release of local calcium signals and subsequent nuclear translocation of transcription factor EB (TFEB), thereby upregulating autophagy-related genes [51]. Furthermore, under conditions of ROS accumulation or endoplasmic reticulum stress, the calcium-dependent kinase CaMKKβ is activated. This activation promotes the assembly of the ULK1 complex through the AMPK/mTORC1 signaling axis, enhancing the expression of key autophagy molecules [47,83,84,85,86]. However, it is crucial to note that the effects of calmodulin and calcium homeostasis on autophagy are complex and context-dependent. Persistent disruption of calcium homeostasis leads to mitochondrial Ca^2+^ overload, which impairs oxidative phosphorylation, generates excessive ROS, and disrupts mitochondrial dynamics, ultimately impairing mitophagy [87]. Additionally, cytoplasmic Ca^2+^ overload can activate calpains, which may disrupt autophagosome-lysosome fusion or impair lysosomal acidification, thereby hindering autophagic flux.

Under pathological conditions, the abnormal regulation of autophagy can manifest as a paradoxical scenario in which the expression of autophagic proteins is upregulated while the autophagic flux is concurrently downregulated. For example, in palmitate-induced MIN6 cells, cytosolic Ca^2+^ overload triggers ER stress (via the PERK/eIF2α/CHOP pathway), promoting apoptosis. However, despite MCU knockdown increasing LC3-II expression, it also results in p62 accumulation, indicating that autophagic flux is compromised [88]. This “autophagy blockage” phenomenon may represent a compensatory response of cells to metabolic stress, as corroborated by findings from other studies [89,90]. Experimental evidence demonstrates that calcium channel blockers or mitochondrial calcium uptake regulators can alleviate this impairment, underscoring the critical role of restoring calcium homeostasis in maintaining autophagic function. Notably, in the context of insulin resistance, β cells with high autophagic flux exhibit heightened sensitivity to glucose-stimulated calcium influx. Conversely, knockout of the autophagy regulatory gene Atg7 abolishes this effect, suggesting that precise modulation of autophagic flux governed by calcium homeostasis could enhance β cell function [91].

#### 3.1.3. Ferroptosis in Pancreatic β-Cells

Ferroptosis is an iron-dependent and lipid peroxidation-driven form of programmed cell death. Its characteristic changes encompass iron metabolism disorders, redox imbalance, and peroxidation of polyunsaturated fatty acids in the cell membrane [92]. Research has revealed that calcium homeostasis dysregulation can promote the accumulation of reactive oxygen species (ROS) through multiple mechanisms, including endoplasmic reticulum stress, mitochondrial dysfunction, and lysosomal damage. This, in turn, forms a vicious cycle with the onset and progression of ferroptosis [93,94].

Iron metabolism exerts a dual regulatory function in pancreatic β-cells. On one hand, via the integration of iron ions into Fe-S clusters mediated by mitochondrial iron transporters (DMT-1, Mfrn1/2), it directly participates in the process of glucose-stimulated insulin secretion (GSIS) [95,96,97]. On the other hand, reactive oxygen species (ROS) generated by the Fenton reaction can serve as second messengers to amplify the insulin-secretion signals [98]. As a result of stimulation by elevated glucose concentrations, inflammatory processes, or environmental toxins, mitochondrial membrane depolarization leads to dysfunction within the electron transport chain. This dysfunction not only impedes ATP synthesis but also initiates a substantial increase in reactive oxygen species (ROS) production. Such conditions may further cause dysregulation of β-cell exosome function, iron-ion overload, and inhibition of GPX4 activity, collectively culminating in pathological changes characteristic of ferroptosis [97,99,100,101]. It is important to acknowledge the intricate relationship between ferroptosis and autophagy. The excessive activation of lipofuscin autophagy and ferritin autophagy can enhance lipid peroxidation through the release of free iron ions, while a deficiency in GPX4 markedly intensifies the cytotoxic effects associated with this mechanism.

**Figure 3:**
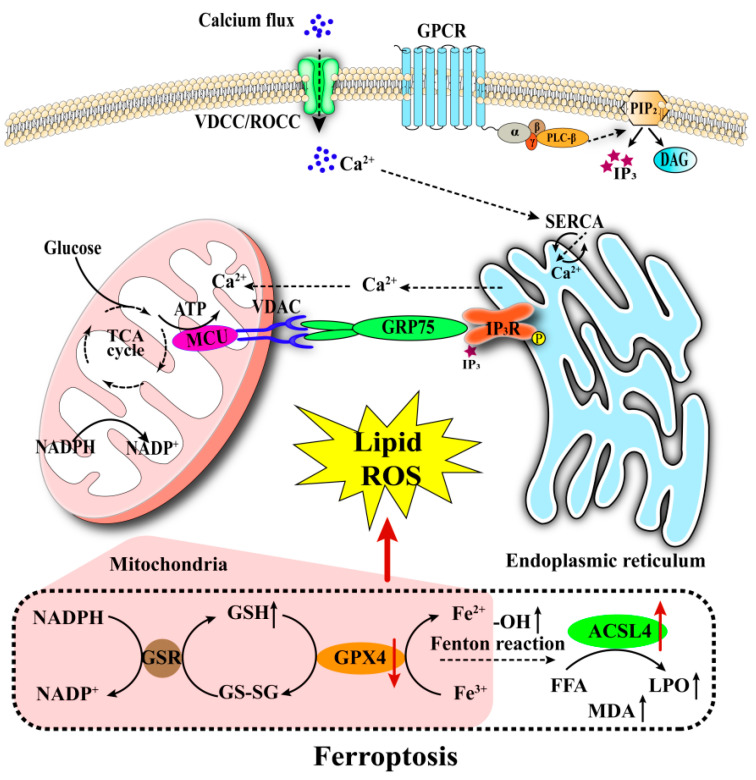
Calcium Dysregulation Drives Ferroptosis. ER stress and calcium release cause mitochondrial dysfunction and glutathione depletion. This leads to iron accumulation and lipid peroxide production via the Fenton reaction. The convergence of calcium imbalance and iron toxicity results in ferroptotic cell death.

Calcium signaling and iron metabolism demonstrate a synergistic influence on the regulation of glucose-stimulated insulin secretion (GSIS) [25,26,97]. These interconnected processes collectively contribute to β-cell damage through the endoplasmic reticulum stress-mitochondrial dysfunction axis (Figure 3). Empirical evidence indicates that the activation of the mitochondrial ROS autophagy lysosome pathway, in conjunction with endoplasmic reticulum stress-related signals, can induce ferroptosis in β-cells. This process is marked by the depletion of glutathione (GSH), the accumulation of lipid peroxidation products, and lipotoxic damage [100,101]. Under glucolipotoxic conditions, endoplasmic reticulum stress results in the depletion of calcium stores, leading to the collapse of mitochondrial membrane potential, the accumulation of mitochondrial ROS (mtROS), and the activation of phospholipases, thereby intensifying the lipid peroxidation process [43,102,103]. While dysregulation of calcium homeostasis and aberrations in iron metabolism can both lead to the accumulation of lipid peroxides, the precise mechanisms underlying their concerted interaction in β-cell injury have yet to be fully elucidated. An in-depth dissection of the interactive regulatory mechanisms within the calcium–iron metabolism network may offer novel therapeutic targets for the protection of β-cells in diabetes.

#### 3.1.4. Pyroptosis in Pancreatic β-Cells

Pyroptosis, a form of programmed cell death facilitated by inflammasomes, is distinguished by the perforation of the cell membrane and the subsequent release of pro-inflammatory cytokines. This process is integral to the pathological progression of diabetes and its associated complications. Calcium ions, serving as a critical intracellular second messenger, play a central regulatory role in the pyroptosis of pancreatic β-cells by precisely modulating the activation of the NLRP3 inflammasome [104,105,106,107,108].

The activation of NLRP3 inflammasome is the core molecular event of pyroptosis, and its triggering factors include multiple stimuli such as ROS accumulation, endoplasmic reticulum stress, lysosomal damage and calcium signal disorder [48,109,110,111]. In the classical pathway, PAMPs/DAMPs promote the assembly of the NLRP3-NEK7-ASC-caspase-1 complex by altering intracellular ion homeostasis (especially K^+^ efflux and Ca^2+^ influx), thereby activating caspase-1 and cleaving GSDMD protein, ultimately leading to the formation of cell membrane pores and the release of IL-1β/IL-18 [112,113,114,115,116]. The non-classical pathway is directly activated by LPS to cleave GSDMD through caspase-4/5/11, and indirectly initiates the classical pyroptosis pathway by secreting IL-1β/IL-18 [117,118,119].

Although direct evidence regarding the regulation of β-cell pyroptosis by calcium homeostasis remains to be further substantiated, calcium homeostasis imbalance associated with endoplasmic reticulum stress may serve as a crucial inducer. Research has indicated that IP3R-mediated calcium release can facilitate hepatocyte pyroptosis by activating the CaMKIIγ/Smad3 pathway [48,120,121]. Under diabetic pathological conditions, endoplasmic reticulum stress induced by high glucose and high lipid over-activates IP3R via the IRE1α/PERK pathway. This leads to the depletion of the endoplasmic reticulum calcium store and cytoplasmic/mitochondrial calcium overload [59,122]. Such a state of calcium overload can trigger the collapse of the mitochondrial membrane potential, an outburst of ROS, and the release of mtDNA. Subsequently, this recruits the assembly of the NLRP3 inflammasome, and IL-1β/IL-18 are released through the caspase-1/GSDMD pathway, ultimately precipitating β-cell pyroptosis [50,123,124].

The mechanisms by which calcium-signal-related pathways regulate pyroptosis have been previously reported. PLCγ1 hydrolyzes PIP2 to generate IP3, which activates IP3R to promote calcium release from the endoplasmic reticulum. This, in turn, enhances the membrane translocation of Gsdmd-N and LPS-induced pyroptosis [63]. In a diabetic heart disease model, CaMKII may promote cardiomyocyte pyroptosis via the TLR4/NLRP3 pathway [64]. Notably, calcium channel blockers can mitigate macrophage pyroptosis and the release of IL-1β by inhibiting the TLR4/NF-κB/NLRP3/GSDMD signaling axis [65]. However, the mechanism by which calcium signals regulate pyroptosis in β-cells remains controversial. Currently, research has mainly focused on calcium homeostasis in muscle cells such as cardiomyocytes and skeletal muscle cells. Given the role of calcium homeostasis imbalance in regulating pyroptosis pathways in other cell types, modulating calcium homeostasis to repair β-cell pyroptosis may represent a novel therapeutic strategy.

### 3.2. Calcium Homeostasis and Insulin Resistance in Diabetes

Insulin resistance is a central pathological aspect in the development and progression of type 2 diabetes. Abundant clinical evidence indicates that it exists prior to the emergence of abnormal β-cell function and can serve as an early predictive marker for diabetes [125,126]. This pathological state is characterized by a reduced sensitivity of peripheral tissues to insulin, concurrently accompanied by compensatory hyperinsulinemia. Significantly, calcium ions, being a crucial second messenger in glucose-stimulated insulin secretion (GSIS), any imbalance in their homeostasis not only impairs β-cell function but also exacerbates peripheral insulin resistance through multiple mechanisms (Table 3) [34,127,128,129].

#### 3.2.1. The Process of Insulin Signaling Transduction

Insulin signaling transduction is a highly intricate cascade reaction. Once insulin binds specifically to the α subunit of the receptor on the surface of target cells, it induces conformational alterations in the β subunit and autophosphorylation of tyrosine residues. This subsequently recruits and phosphorylates insulin receptor substrate (IRS) proteins [131,132]. The phosphorylated IRS activates phosphatidylinositol 3-kinase (PI3K) via its SH2 domain, catalyzing the transformation of phosphatidylinositol 4,5-bisphosphate (PIP2) into phosphatidylinositol 3,4,5 -trisphosphate (PIP3). PIP3 then activates protein kinase B (Akt/PKB) through a phosphoinositide-dependent kinase 1 (PDK1) dependent pathway. The activated Akt, on one hand, facilitates the translocation of the glucose transporter GLUT4 to the cell membrane, thereby enhancing glucose uptake. On the other hand, it inhibits gluconeogenesis and promotes glycogen synthesis by regulating downstream effector molecules such as glycogen synthase kinase-3 (GSK-3).

#### 3.2.2. Insulin Resistance in Target Organs

In hepatic tissues, disruptions in calcium homeostasis substantially impair the PI3K-Akt signaling pathway. Empirical evidence indicates that calcium overload can decrease the phosphorylation level of insulin-stimulated Akt by 50% and reduce glucose uptake efficiency by 60% [127].

In the context of obesity, lipid accumulation in hepatocytes leads to an increased cytoplasmic Ca^2+^ concentration. The resulting Ca^2+^-phosphatidylinositol complex competitively inhibits the membrane localization of the PH domain of Akt, thereby directly disrupting insulin signal transduction. Inhibition of SOCE channels has been shown to mitigate these effects [34,128].

In skeletal muscle, the influence of calcium overload on insulin resistance demonstrates a bidirectional regulatory nature. A high-fat diet can induce a burst of mitochondrial reactive oxygen species (ROS), leading to the release of mitochondrial calcium through the opening of the mitochondrial permeability transition pore (mPTP). This release activates calcium-dependent proteases, such as calpain, which disrupt the GLUT4 transport system [133]. Conversely, the CaMKII-AMPK-PKC pathway, activated by calcium influx, can facilitate the translocation of GLUT4 to the membrane, thereby enhancing insulin sensitivity [49]. It is important to note that the overactivation of a different isoform of CaMKII, specifically CaMK2, downregulates the expression of insulin receptors in adipocytes, resulting in impaired insulin signal transduction. This seemingly contradictory phenomenon may be attributed to the specific activation of distinct calcium signaling pathways.

In adipose tissue, the development of insulin resistance is driven by the complex interactions between intracellular and extracellular calcium signaling. Insufficient calcium intake can enhance calcium influx into adipocytes through a vitamin D-dependent mechanism, thereby promoting lipogenesis [134]. In obese models, increased lipid synthesis significantly inhibits the activity of SERCA, leading to a disturbance in endoplasmic reticulum calcium homeostasis. This disruption subsequently induces serine phosphorylation of insulin receptor substrate 1 (IRS1) via the c-Jun N-terminal kinase (JNK)/inhibitor of nuclear factor kappa-B kinase subunit beta (IKKβ) pathway, thereby impairing insulin signal transduction [130,135,136]. Additionally, the activation of calcineurin enhances the expression of lipid-synthesizing enzymes. Lipid metabolites, such as ceramide, produced by these enzymes, inhibit the activation of protein kinase B (Akt) through the protein kinase C (PKC)/protein phosphatase 2A (PP2A) pathway, thereby establishing a self-perpetuating cycle of “calcium imbalance–lipid accumulation–insulin resistance” [137,138].

Although numerous fundamental research studies have demonstrated the substantial impact of calcium homeostasis on insulin resistance, the role of calcium channel blockers in clinical practice remains contentious. Some studies suggest that nifedipine improves insulin resistance by indirectly enhancing the activity of glucose transporter 4 (GLUT-4) through the activation of protein phosphatase 1 (PP-1) [129]. Conversely, a separate randomized double-blind controlled trial found that calcium channel blockers did not significantly affect insulin sensitivity [139]. This inconsistency may arise from the complex pathogenesis of insulin resistance. Calcium signaling constitutes only one element of this intricate network, and other factors, such as post-receptor signaling pathways, may also influence the overall therapeutic outcome.

## 4. Calcium Homeostasis and Gout

The primary pathological mechanism underlying gout is the inflammatory cascade initiated by the deposition of monosodium urate (MSU) crystals. Recent studies have elucidated the critical regulatory role of disruptions in calcium homeostasis in this process [38,140]. While the role of MSU crystals in triggering inflammation is well-established, aberrant calcium signal transduction also plays a significant role in the pathological progression of gout. This occurs through its impact on immune cell function, activation of the NLRP3 inflammasome, and induction of insulin resistance.

### 4.1. Abnormal Calcium Signaling in Immune Cells

#### 4.1.1. T Cells and Calcium Homeostasis

The involvement of T-cell-mediated immune dysregulation in gouty arthritis is of considerable importance, with calcium signaling disruption playing a pivotal role [141]. Clinical investigations have demonstrated that patients with early-onset gout exhibit a marked imbalance in the Th17/Treg cell ratio, characterized by an expansion of Th17 cells and a reduction in Treg cells [142]. This immune dysregulation leads to the excessive secretion of pro-inflammatory cytokines, such as interleukin-17 (IL-17), thereby promoting tophi formation and disease progression. Mechanistically, calcium oscillations generated upon T-cell receptor activation play a crucial role in regulating Th1/Th17 cell differentiation and cytokine secretion through the calcineurin-nuclear factor of activated T-cells (NFAT) signaling pathway [143,144]. Importantly, a high-calcium microenvironment can enhance the expression of key transcription factors, including T-box expressed in T cells (T-bet) and retinoid-related orphan receptor gamma t (RORγt), further facilitating the polarization of Th1/Th17 cells [145]. Therefore, targeting calcium-dependent immune regulation may represent a novel therapeutic strategy for gout.

#### 4.1.2. Macrophages and Calcium Homeostasis

The activation of the NLRP3 inflammasome, facilitated by the dysregulation of calcium homeostasis in macrophages, represents a critical component of inflammation associated with gout [146,147]. Throughout the pathogenesis of gout, disruptions in calcium signaling activate the NLRP3 inflammasome via multiple pathways. This activation triggers an inflammatory cascade, culminating in the characteristic clinical symptoms of acute gouty arthritis.

The disturbance of calcium homeostasis is a crucial factor in initiating NLRP3 inflammasome activation. Studies have demonstrated that the deposition of monosodium urate (MSU) crystals impairs mitochondrial function, leading to the accumulation of reactive oxygen species (ROS) and the creation of a pro-inflammatory microenvironment, including hypoxia, which subsequently induces endoplasmic reticulum (ER) stress. In particular, the c-Jun N-terminal kinase (JNK)-mediated hyperphosphorylation of inositol 1,4,5-trisphosphate receptors (IP3Rs) leads to enhanced calcium efflux from the endoplasmic reticulum (ER). Concurrently, mitochondria excessively absorb calcium ions through the voltage-dependent anion channel 1 (VDAC1)-glucose-regulated protein 75 (GRP75)-mitochondrial calcium uniporter (MCU) complex, resulting in the phenomenon known as “calcium signal appropriation.” Therefore, ER stress induced by calcium dysregulation is not exclusive to the metabolic stress in β-cells (explored in Section 3.1.1), ER stress also emerges in immune cells during gouty inflammation, triggered by MSU crystals and oxidative stress, indicating its role as a convergent node in the pathology of both diseases. This disruption of calcium signaling not only compromises the protein-folding capacity of the ER but also activates the nuclear factor-κB (NF-κB) signaling pathway via mitochondrial dysfunction and oxidative stress, thereby facilitating the activation of the NLRP3 inflammasome [148,149,150]. Upon interaction with the macrophage membrane, monosodium urate (MSU) crystals are internalized and transported to lysosomes, leading to lysosomal membrane destabilization and the subsequent release of adenosine triphosphate (ATP). The released adenosine triphosphate (ATP) activates the P2X7 receptor ion channel, thereby facilitating the influx of Ca^2+^. The inflammatory cascade reaction triggered by the disruption of intracellular calcium homeostasis promotes the assembly of the NLRP3 inflammasome. This, in turn, mediates the maturation and release of key inflammatory cytokines such as IL-1β and IL-8 [151,152,153,154,155]. The ensuing cycle of mitochondrial dysfunction and oxidative stress, precipitated by endoplasmic reticulum (ER) calcium depletion, further intensifies the inflammatory response [156,157]. This calcium-dependent NLRP3 activation in macrophages, central to acute gouty arthritis, is mechanistically paralleled by similar processes in pancreatic β-cells (as discussed in Section 3.1.4) and contributes to the systemic chronic low-grade inflammation.

Additionally, mitochondrial calcium overload in macrophages prompts the opening of the mitochondrial permeability transition pore (mPTP), a process more likely to induce pyroptosis rather than classical apoptosis [158,159]. This preferential shift in cell death modality may constitute a critical feature of acute gout inflammation and provides a novel perspective for understanding the pathological mechanisms underlying gout.

### 4.2. Insulin Resistance and Imbalance of Calcium Homeostasis in Gout

A bidirectional causal relationship between insulin resistance and gout has been identified. Genetic studies have revealed a significant positive correlation between insulin resistance and an increased risk of developing gout [160]. Clinical observations have corroborated these findings, indicating that the severity of gout is closely associated with markers of insulin resistance [161]. At the molecular level, uric acid has been shown to directly interfere with the insulin signaling pathway by promoting the binding of ENPP1 to the insulin receptor (IR), an effect that occurs independently of the oxidative stress and inflammatory pathways typically activated by uric acid [15]. These findings imply that gout and insulin resistance may form a mutually reinforcing vicious cycle.

The disruption of calcium homeostasis represents a critical pathological link between insulin resistance and gout. Current evidence suggests that the calcium ion signaling system plays a role in the regulation of insulin signal transduction through various mechanisms. Specifically, intracellular calcium overload can hinder IRS tyrosine phosphorylation via endoplasmic reticulum stress. Additionally, abnormal activation of the calcineurin pathway can significantly diminish the efficiency of GLUT4 membrane translocation [49,127,129]. Furthermore, dysregulated expression of endoplasmic reticulum calcium channel genes can exacerbate the endoplasmic reticulum stress response and impair pancreatic β-cell function [162]. Collectively, these mechanisms contribute to a reduction in insulin sensitivity, resulting in insulin resistance and subsequent hyperinsulinemia. Importantly, elevated insulin levels can inhibit uric acid excretion by upregulating the activity of the URAT1 transporter [163]. Conversely, disturbances in uric acid metabolism can further aggravate insulin resistance through oxidative stress and other pathways [15], thereby establishing a positive feedback regulatory loop.

The disruption of calcium homeostasis has been implicated in the onset of gouty inflammation. Studies have demonstrated that calcium influx, triggered by factors such as metabolic abnormalities (including hyperglycemia and hyperlipidemia) and oxidative stress, can directly activate the NLRP3 inflammasome and facilitate the release of IL-1β [117,136,148,164]. This mechanism not only exacerbates insulin resistance but also precipitates acute gouty arthritis. Notably, the overexpression of RIPK3 has been shown to increase cytoplasmic Ca^2+^ and reactive oxygen species (ROS) levels through the endoplasmic reticulum stress pathway. This, in turn, upregulates the expression of xanthine oxidase (XO), thereby accelerating uric acid production [165]. These findings elucidate the pivotal role of aberrant calcium signaling in the molecular interplay between insulin resistance and gout.

## 5. The Association Between Intracellular Calcium Homeostasis and Comorbid Gout in Type 2 Diabetes

A significant comorbidity association exists between gout and type 2 diabetes [5]. Epidemiological evidence suggests that the prevalence of gout among individuals with type 2 diabetes is 3.8 times higher than in the general population [166]. Multivariate adjustment analyses reveal that, even after controlling for confounding factors such as obesity and hypertension, gout is associated with a 17% to 47% increased risk of developing type 2 diabetes [167,168]. This notable comorbidity phenomenon involves multiple pathological mechanisms, including insulin resistance, chronic low-grade inflammation, dyslipidemia, and oxidative stress. Importantly, dysregulation of calcium homeostasis may serve as a crucial regulatory nexus, contributing not only to the initiation of the disease but also to the amplification of metabolic disruption signals, thereby facilitating the co-progression of type 2 diabetes and gout.

Beyond the parallel pathophysiological roles of calcium dysregulation in type 2 diabetes and gout individually, its function as a central amplifier in their comorbidity can be conceptualized through three interconnected themes: a self-reinforcing calcium cycle, a central inflammatory nexus, and a novel perspective on β-cell death. First, a self-reinforcing calcium cycle is established: calcium-triggered β-cell dysfunction and peripheral insulin resistance (as detailed in Section 3) lead to hyperinsulinemia and hyperglycemia, which promote hyperuricemia (as outlined in Section 2). The resulting inflammatory milieu, in turn, further disrupts calcium homeostasis in immune cells and metabolic tissues (as explored in Section 4), creating a feed-forward loop that amplifies the entire pathology. Second, calcium acts as the central inflammatory nexus, serving as a common upstream signal that translates diverse metabolic insults (e.g., hyperglycemia, free fatty acids, MSU crystals) into the shared outcome of NLRP3 inflammasome activation, thereby fueling the chronic inflammation characteristic of both diseases. Finally, within the pancreatic islet, calcium dysregulation provides a novel perspective on β-cell demise by orchestrating the crosstalk and temporal sequence between programmed cell death pathway (pyroptosis), which may be crucial for understanding the accelerated loss of insulin secretion in the comorbid state. The following sections will elaborate on the evidence for this integrative framework.

### 5.1. Linking Effects of Insulin Resistance

Research has demonstrated that the onset of insulin resistance occurs prior to the clinical diagnosis of type 2 diabetes [125]. In the initial stage of elevated blood glucose levels, glucose is transported into β-cells via the GLUT2 transporter. This is followed by an influx of Ca^2+^, which triggers the glucose-stimulated insulin secretion (GSIS) process [25,26]. In response to metabolic demands, pancreatic β-cells compensate by augmenting the synthesis of pro-insulin. This increased synthesis imposes a significant burden on the endoplasmic reticulum (ER), leading to the activation of the unfolded protein response (UPR) and the induction of endoplasmic reticulum stress. The aberrant activation of inositol 1,4,5-trisphosphate (IP3) receptors within the endoplasmic reticulum precipitates increased calcium leakage. Concurrently, mitochondria excessively sequester calcium ions via the VDAC1-GRP75-MCU complex, culminating in mitochondrial dysfunction. This intracellular calcium overload exacerbates stress in both the endoplasmic reticulum and mitochondria, facilitates the accumulation of reactive oxygen species (ROS), and triggers pro-inflammatory signaling pathways, including nuclear factor-κB (NF-κB) [148,149,150]. Collectively, these pathological changes impede the phosphorylation of insulin receptor substrate (IRS) in hepatic, muscular, and adipose tissues, thereby disrupting the normal function of the phosphatidylinositol 3-kinase/protein kinase B (PI3K/Akt) and mitogen-activated protein kinase (MAPK) signaling pathways. Consequently, this impairment diminishes the glucose uptake capacity mediated by glucose transporter 4 (GLUT4), perpetuating a deleterious cycle of insulin resistance [129,135,169].

Hyperinsulinemia resulting from insulin resistance can substantially activate the Na^+^-H^+^ exchanger in the proximal convoluted tubules of the kidneys and enhance the expression of the URAT1 transporter. This process facilitates the reabsorption of uric acid while inhibiting its excretion [8,9]. Concurrently, a hyperglycemic state triggers the polyol pathway, accelerating fructose metabolism and depleting adenosine triphosphate (ATP). This degradation process further elevates the production of endogenous uric acid [11]. Elevated serum uric acid levels promote the phosphorylation of insulin receptor substrate 2 (IRS2) at serine 731 and inhibit the phosphorylation of protein kinase B (Akt) at serine 473 via oxidative stress mechanisms, thereby impairing insulin signaling [170]. Additionally, increased levels of retinol-binding protein 4 (RBP4) associated with hyperuricemia (HUA) can specifically inhibit the IRS/phosphatidylinositol 3-kinase (PI3K)/Akt pathway in adipocytes, further exacerbating insulin resistance [171]. These interconnected molecular mechanisms collectively contribute to the pathological progression of gout comorbid with type 2 diabetes.

It is noteworthy that hyperglycemic and hyperuricemic conditions can independently trigger endoplasmic reticulum (ER) stress [172,173]. Dysregulation of calcium homeostasis is critically involved in the regulation of ER stress and insulin signaling pathways [34,40]. Based on these observations, we hypothesize that ER stress-induced disruption of calcium homeostasis may represent a significant molecular mechanism contributing to the onset of insulin resistance, which may subsequently result in the pathological manifestations of gout in individuals with type 2 diabetes.

### 5.2. The Amplification of Inflammatory Signals

**Figure 4:**
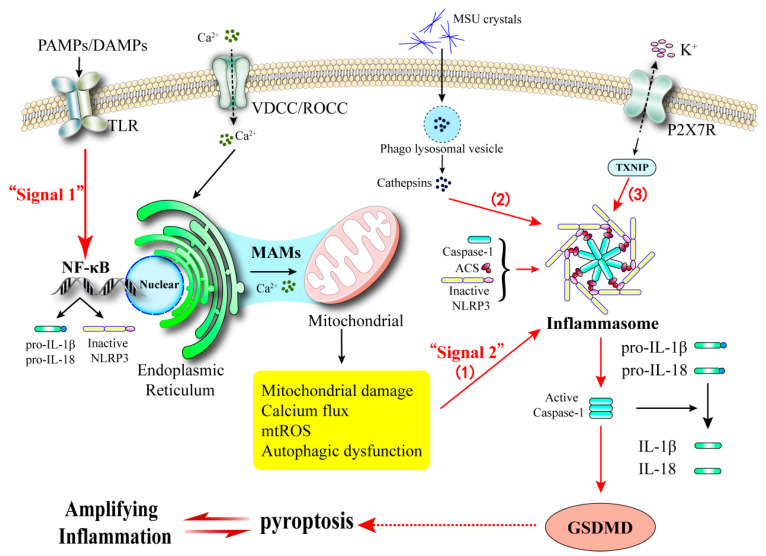
Calcium Influx Triggers Pyroptosis via the NLRP3 Inflammasome. Danger signals (PAMPs/DAMPs) prime the NF-κB pathway to express NLRP3 and pro-cytokines. Calcium overload serves as a key signal for NLRP3 inflammasome assembly. Inflammasome activation cleaves GSDMD, forming pores in the membrane and executing pyroptosis.

The secretion of interleukin-1β (IL-1β), mediated by the NLRP3 inflammasome, plays a pivotal regulatory role in the innate immune responses associated with type 2 diabetes and gout. As previously elaborated, calcium dysregulation is a key trigger for NLRP3 activation in both pancreatic β-cells (Section 3.1.4) and immune cells such as macrophages in gout (Section 4.1.2). Here, we focus on how this shared mechanism creates an amplified inflammatory signal within the comorbid state. Calcium ions, functioning as second messengers, are essential for the activation of this pathway (Figure 4). Current research suggests that elevated uric acid levels can lead to the precipitation of monosodium urate (MSU) crystals. This process subsequently induces the synthesis of oxidized mitochondrial DNA (mtDNA), increases the production of reactive oxygen species (ROS), exacerbates impairments in mitochondrial oxidative phosphorylation, and results in oxygen depletion, ultimately disrupting cellular calcium homeostasis [174,175]. The influx of intracellular calcium ions (Ca^2+^) can effectively activate the MAPK/NF-κB signaling pathway, which promotes the polarization of macrophages towards a pro-inflammatory phenotype (M1 type), thereby significantly enhancing their phagocytic capabilities [176]. The polarization of M1-type macrophages induces K^+^ efflux and Ca^2+^ influx, thereby establishing a positive feedback loop that exacerbates calcium homeostasis imbalance. This process facilitates the assembly of the NLRP3 inflammasome and the activation of Caspase-1, accompanied by the cleavage of the gasdermin D (GSDMD) protein, which leads to pyroptosis. Consequently, there is a substantial release of pro-inflammatory cytokines, such as IL-1β and IL-18. Concurrently, the expression of inflammatory mediators, including nitric oxide (NO) and tumor necrosis factor-α (TNF-α), is upregulated [112,113,114,115,116]. Ultimately, this sequence of events establishes a critical inflammatory foundation conducive to the development of gout in the context of diabetes.

Under physiological conditions, the limited activation of inflammasomes maintains a low concentration of IL-1β, which can appropriately stimulate insulin secretion. However, overexpression of IL-1β may induce chronic, low-grade inflammation within pancreatic islets [177,178]. This inflammatory state impairs the glucose-stimulated calcium influx (GSCI) capability of β cells, ultimately leading to a reduction in insulin secretion. Additionally, IL-1β can downregulate the expression of genes associated with β-cell maturation and disrupt intracellular calcium homeostasis [179,180,181,182,183]. The disturbance in calcium homeostasis activates phospholipase C (PLC) and protein kinase C ε (PKCε), which in turn amplify the NF-κB and NLRP3/GSDMD inflammatory pathways. This cascade promotes the release of cytokines, such as IL-1β, and exacerbates β-cell pyroptosis. More importantly, dysregulated calcium signaling in β cells can also activate the death receptor pathway and induce endoplasmic reticulum stress, leading to β-cell apoptosis [43,173]. Through the dual mechanisms of apoptosis and pyroptosis, there is a reduction in both the number and functionality of β cells. This process represents a critical step in the pathogenesis of gout in the context of diabetes.

A diverse array of chemokines facilitates the differentiation and aggregation of inflammatory cells, which subsequently release substantial quantities of pro-inflammatory factors. These factors perpetually activate the immune response and enhance downstream inflammatory reactions, thereby establishing a positive feedback loop [184,185]. This cascade not only exacerbates damage to pancreatic β-cells but also promotes chronic inflammation in insulin-sensitive tissues, including adipose tissue, the liver, and skeletal muscle. As a result, it further disrupts insulin signal transduction and exacerbates insulin resistance. The interplay between calcium homeostasis imbalance and inflammatory pathways can engender a pro-inflammatory microenvironment, which intensifies endoplasmic reticulum stress and mitochondrial damage. This ultimately leads to pan-apoptosis or autophagy of β-cells [6,186,187]. Consequently, this process emerges as a pathogenic factor in the development of gout complicated by type 2 diabetes.

### 5.3. Linking β-Cell Death Networks to Gouty Inflammation via Calcium Signaling

Beyond the direct inflammatory assault, the dysregulated calcium signaling within β-cells themselves instigates a multifaceted cell death network, offering a novel perspective on their progressive decline in the context of comorbid gout [53,57,58,59]. While pyroptosis, with its inflammatory character, forms a direct bridge with the systemic inflammatory nexus described above, it is likely not the sole executor. We posit that calcium overload orchestrates a dynamic interplay between multiple programmed death pathways: calcium-induced mitochondrial permeability commits cells to the apoptotic pathway [74,75,76,77]; simultaneously, calcium-mediated ER stress and ROS production can trigger ferroptosis, an iron-dependent death characterized by lipid peroxidation [43,93,103]; as detailed earlier, calcium also activates the NLRP3 inflammasome within the β-cell, leading to pyroptosis and the release of more IL-1β [50,104,105,106,107,108].

Critically, these pathways do not operate in isolation. For instance, apoptotic signaling can inhibit pyroptosis, while components of autophagy (dysregulated by calcium) can promote ferroptosis [51,60,100]. The crosstalk and temporal sequence of these calcium-driven death modalities—determining whether a β-cell dies quietly (apoptosis) or incites an inflammatory riot (pyroptosis)—may be a key determinant in the rate of β-cell mass loss and the intensity of local islet inflammation.

This accelerated β-cell demise, driven by the calcium-dependent death network, has direct implications for the comorbidity with gout. A more rapid decline in β-cell function would exacerbate hyperinsulinemia and hyperglycemia, which are key drivers of hyperuricemia (as established in Section 5.1) [8,9,11]. Consequently, the calcium-mediated ‘inflammatory riot’ (pyroptosis) within the islet not only damages the pancreas locally but also systemically fuels the metabolic dysregulation that promotes and worsens gout, creating a vicious cycle between pancreatic failure and gouty inflammation. Therefore, targeting the master regulator, calcium homeostasis, could simultaneously protect β-cells and mitigate a key driver of gout progression [41,45].

## 6. Therapeutic Perspectives and Challenges

The compelling evidence linking calcium dysregulation to the type 2 diabetes–gout axis naturally prompts the consideration of calcium channel modulators as potential therapeutic agents. Preclinical studies offer promising leads: SERCA activators like CDN1163 have been shown to improve ER stress and insulin sensitivity in obese mouse models [41], while inhibitors of store-operated calcium entry (SOCE) can ameliorate hepatic insulin resistance [34]. Furthermore, common calcium channel blockers (CCBs), such as nifedipine, have been reported to indirectly enhance GLUT4 activity and improve insulin sensitivity in some studies [129].

However, the translation of these findings into clinical practice faces substantial and multifaceted challenges. The lack of tissue specificity of first-generation modulators poses a significant risk of off-target effects, given the ubiquitous role of calcium signaling. This critical limitation is exemplified by the conflicting clinical data on CCBs; while some studies suggest benefits, others, including randomized controlled trials, have failed to demonstrate a significant improvement in insulin sensitivity [139]. The complex and often opposing roles of calcium signals in different tissues further complicate therapeutic targeting. For instance, the activation of specific calcium signaling pathways (e.g., certain CaMKII isoforms) may enhance insulin sensitivity in one tissue while promoting resistance in another [49,130].

Most importantly, there is a conspicuous absence of large-scale, high-quality clinical trials specifically designed to test the efficacy and safety of calcium-focused therapies in patients with type 2 diabetes and comorbid gout. The current evidence base is insufficient to clarify the exact therapeutic potential of modulating calcium homeostasis in this specific patient population. These unresolved issues—ranging from fundamental biological complexity to a lack of clinical validation—constitute the core of the current controversy and highlight the significant gap between mechanistic promise and clinical application.

Therefore, future efforts must bifurcate: first, to focus on designing next-generation, tissue-restricted modulators that can precisely target pathological calcium signaling without disrupting physiological functions. Second, well-designed clinical studies are urgently needed to validate whether correcting calcium homeostasis can safely and effectively break the vicious cycle linking hyperinsulinemia, hyperuricemia, and chronic inflammation in this high-risk patient population.

## 7. Conclusions

In conclusion, this review definitively establishes intracellular calcium dysregulation as a pivotal mechanism and a unifying pathological nexus linking the pathogenesis of type 2 diabetes and gout. We have synthesized evidence demonstrating that calcium homeostasis imbalance acts as a central amplifier, driving β-cell injury through ER stress, mitochondrial dysfunction, and multiple programmed cell death pathways (apoptosis, autophagy, ferroptosis, pyroptosis), while concurrently exacerbating peripheral insulin resistance and activating the NLRP3 inflammasome to fuel chronic inflammation. This integrated perspective moves beyond a simple compilation of known mechanisms and positions calcium signaling as a key orchestrator of the comorbidity.

Despite these advances, critical knowledge gaps remain. The temporal dynamics of calcium dysregulation during the progression from isolated type 2 diabetes to comorbid gout are poorly understood. Furthermore, the precise crosstalk between calcium-dependent cell death modalities in β-cells and immune cells represents a complex, underexplored area. The tissue-specificity of calcium channels necessitates the development of targeted modulators to avoid off-target effects, a significant current limitation.

Future research should therefore prioritize several key directions: (1) developing tissue-specific calcium channel modulators to minimize systemic side effects; (2) conducting longitudinal clinical studies to validate calcium homeostasis biomarkers as predictors for gout onset in diabetic populations; (3) employing single-cell technologies to elucidate the precise interplay between calcium, iron metabolism, and inflammasome activation in different cell types. Addressing these specific avenues is not merely an academic exercise but holds promising potential for overcoming the therapeutic challenges in managing type 2 diabetes complicated by gout, ultimately paving the way for novel, mechanism-based interventions.

## Figures and Tables

**Table 1:** Proteins regulating calcium homeostasis.

Name/Location	Function	Main Regulators	References
SERCA (ER membrane)	Ca^2+^ uptake from cytosol to ER lumen	Thapsigargin (inhibitor); CDN1163 (activators); Aged garlic extract (activators);	[40,41,42,43]
IP3R (ER membrane)	Ca^2+^ release from ER to cytosol	Xestospongin C (inhibitor); ryanodine (inhibitor); 2-APB (inhibitor); carbachol (activators); IP3 (activators);	[43,44]
RyR (ER membrane)	Ca^2+^ release from ER to cytosol	Dantrolene (inhibitor); High Ca^2+^ (inhibitor); Low Ca^2+^ (activator); Caffeine (activator);	[43,44]
STIM (ER membrane)	Ca^2+^ entry from extracellular space to cytosol	Ca^2+^ depletion (activator); Low 2APB (activator); SKF96365 (inhibitor); Lupenone (inhibitor); BTP2 (inhibitor);	[20,45,46]
Orai (Plasma Membrane)	Coupled with STIM, Ca^2+^ entry from extracellular milieu to cytosol	A chaperone complex (Regulating agent); BTP2 (inhibitor);	[20,46]
CaMKII (Cytoplasm)	Regulated by the interaction between Ca^2+^ and calmodulin	Ionomycin (activator);	[45,47,48,49]
VDAC (Mitochondrial outer membrane)	Forms a channel in the outer mitochondrial membrane and coupled to MCU to allow Ca^2+^ diffusion	BD1047 (Regulating agent);	[24,46,50]
MCU (Mitochondrial inner membrane)	Ca^2+^ uptake into the mitochondria	Ruthenium Red (inhibitor) BAPTA-AM (Regulating agent);	[24,46,50]
TRPML1 (Lysosome)	Mediating lysosomal Ca^2+^ efflux and promoting autophagy	PI (3,5) P_2_ (activator)	[51]

**Table 2:** Mechanisms by which different intracellular calcium channels influence cell injury.

Forms of Injury	Calcium Channels (Activation/Inhibition)	Mechanisms	References
Apoptosis	SERCA (inhibition);	Improving the calcium storage in the endoplasmic reticulum and alleviating endoplasmic reticulum stress.	[40,42,43,53]
	IP3R, RyR (activation);	Resulting in depletion of calcium ions in the endoplasmic reticulum, triggering endoplasmic reticulum stress.	[43,44,62]
	SOCE (activation)	Increasing the intracellular calcium ion concentration, causing endoplasmic reticulum stress.	[45]
Autophagy	TRPML1 (activation);	Mediating the release of calcium ions from lysosomes, activating Calcineurin, and inducing autophagy.	[51]
	CRAC (activation);	Mediating the inward flow of extracellular calcium ions and promoting autophagy.	[51]
Pyroptosis	IP3R (activation)	PLCγ1 indirectly activating IP3R to release endoplasmic reticulum calcium to induce pyroptosis.	[63]
	CaMKII (activation)	Promoting the assembly of the NLRP3 inflammasome and activating pyroptosis.	[64]
	L-type calcium channel (inhibition)	Reducing the intracellular calcium ion concentration and inhibiting the activation of the NLRP3 inflammasome.	[65]

**Table 3:** Mechanisms by which different calcium channels affect insulin resistance.

Calcium Channels	Animals/Cells	Intervention Reagents	Treatment Time	Disease Type	Mechanisms	References
SERCA (inhibitor)	ob/ob mice	CDN1163	5 days	Insulin resistance and prediabetes	Activating Ca^2+^-ATPase activity and improving endoplasmic reticulum stress	[41]
SOCE (activator)	Male C57BL/6 mice HepG2 cells	Candesartan Azilsartan, candesartan,	21 days 16 h	Obesity; Insulin resistance	Mediated calcium influx, inhibition of AKT phosphorylation	[34]
CaMKII (inhibitor)	L6-GLUT4myc cells	Lonomycin	48 h	Muscle Insulin resistance	Activating AMPK-PKC phosphorylation pathway to enhance GLUT4 exocytosis and endocytosis	[49]
CAMK2 (activator)	Ai-CAMK2 KO mice; OP9 cells	Tamoxifen KN93	5 days 1 h	Obesity; Insulin resistance	CAMK2 activation decreases the number of insulin receptors (INSR)	[130]

## Data Availability

No new data were created or analyzed in this study.
